# Congenital scoliosis in monozygotic twins: case report and review of possible factors contributing to its development

**DOI:** 10.1186/1748-7161-3-17

**Published:** 2008-11-18

**Authors:** Angelos Kaspiris, Theodoros B Grivas, Hans-Rudolf Weiss

**Affiliations:** 1 Department of Trauma and Orthopaedics, "Thriasio" General Hospital - NHS, G. Gennimata Av. 19600, Magoula, Attica, Greece; 2 Asklepios Katharina Schroth, Spinal Deformities Rehabilitation Centre, Korczakstrasse 2, D – 55566 Bad Sobernheim, Germany

## Abstract

**Background:**

The exact etiology of congenital scoliosis remains unknown as yet. It seems that its development may be influenced by both genetic predisposition and environmental factors, at varying degrees. International bibliography features few cases of monozygotic twins with congenital scoliosis. The aim of this study is to report a case in monozygotic twins and review the literature relating to the description of similar cases as well as the pathophysiological mechanism involved in its development.

**Methods:**

Clinical examination and simple X-rays revealed scoliosis of differing degrees and types in male monozygotic twins with moderate mental retardation and dyslalia.

**Results:**

Congenital scoliosis identified in both twins. In the first, this was manifested as left thoracic scoliosis, with Cobb angle of 34 degrees while in the second as left thoracolumbar scoliosis with Cobb angle of 10 degrees. Both were found to suffer from incarcerated hemivertebrae.

**Conclusion:**

According to both its clinical identification and severity and to its course, not only the genetic but the environmental factors seem to play a leading role in the appearance of the condition.

## Background

Congenital scoliosis is the abnormal development of the spine resulting in combination of missing portion, partial formation, or lack of separation of the vertebras [1]. The vertebral malformations spanned the length of the entire spine and were classified as butterfly vertebrae, segmentation defect, hypoplasia and hemivertebrae. Butterfly vertebrae were defined by the presence of sagittal cleft [1]. Congenital scoliosis is often accompanied by other dysplastic anomalies and may affect multiple organ systems. It is characteristically described that its clinical manifestation may be linked to around seventy different syndromes [2-5]. Despite the large number of epidemiological and clinical studies and extended research at molecular level, its etiology remains unclear. This seems to implicate both genetic and environmental factors. Our paper reports a case of congenital scoliosis in monozygotic twins, to be added to the very few cases found in international literature.

## Case report

**Figure 1:**
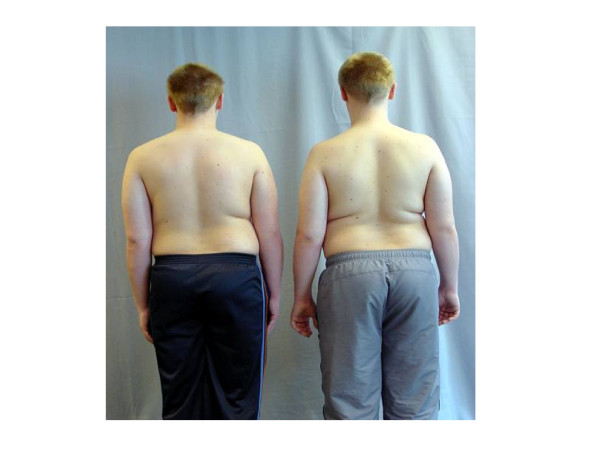
Through the clinical evaluation of the twins in standing position the observation has revealed a slight shoulder asymmetry that accompanied by a slight scapular and waist asymmetry in the first and a moderate asymmetry of the above somatic areas in the second.

The case involves two male monozygotic twins, born in May 1992, following a full-term pregnancy. There was no history of consanguinity of the parents. The mother's history does not include exposure to smoke, toxins or drugs during pregnancy and the family history does not feature any cases of congenital spinal malformations. Congenital scoliosis was diagnosed at the age of 11. Both twins are 1.71 m tall and weight 88 and 81 kg respectively. Moderate thoracic curves were revealed in both upon clinical examination (Figure 1). No pathological findings were noted on the skin or subcutaneous tissue, while the examination results for the remaining systems were within normal limits. No accompanying musculoskeletal abnormalities or neurological symptomatology were noted, apart from moderate mental retardation and dyslalia in both twins.

**Figure 2:**
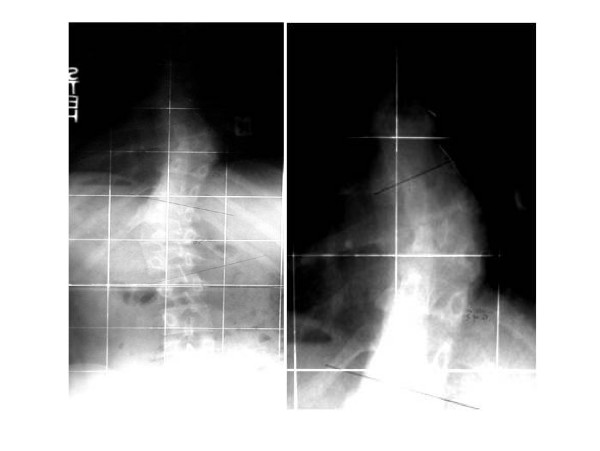
Anterior posterior plain X-ray of the first twin at the age of 11 years demonstrates a left thoracic scoliosis with an upper curve from Th6 – Th10 and a semiincarcarated hemivertebra and a lower curve Th10 – L2 with a Cobb angle of 34°.

**Figure 3:**
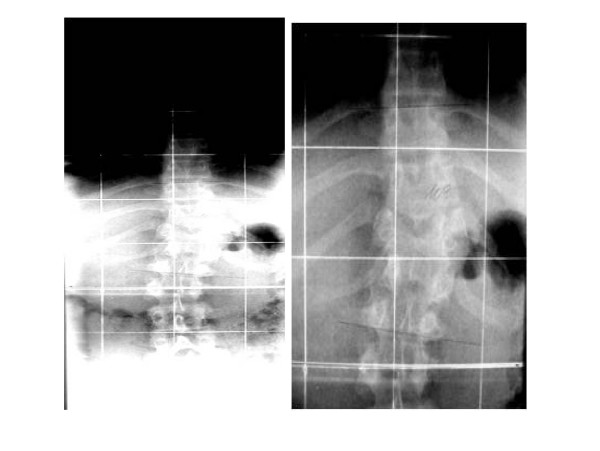
Child two was first seen at the age of 11 and had a left thoraco lumbar scoliosis with a curve from Th9 – L1 and a Cobb angle of 10°.

During the anterior-posterior spine X-ray examination we identified at the first child a left scoliosis with a Cobb angle of 34°. The upper curve is between Th6 and Th10 with semiincarcerated hemivertebrae while the lower curve is between T10 and L2 (Figure 2). The second twin has a left thoracolumbar scoliosis (Th9 – L1) with a Cobb angle of 10° (Figure 3). The X-ray examination of the pelvis showed this to be Risser grade 3, without further development during the last semester of the examination. The scoliosis has manifested a very limited gradual progression since the time of diagnosis. Given the moderate nature of the condition and its benign prognosis, neither therapeutic treatment has been initiated nor further MRI study required.

A written informed consent was obtained from the patients for publication of this case report and accompanying images. A copy of the written consent is available for review by the Editor – in chief of this journal.

## Discussion

The frequency of congenital scoliosis is approximately 0.5, or 1 in 1,000 births. The etiological factors involved in its development are unknown. Basic research has implicated various environmental factors, such as the exposure of the pregnant mother to carbon monoxide, the consumption of alcohol [6,7] or the administration of antiepileptic agents, such as valproic acid [6,8,9] and Dilantin [6]. Other factors that seem to participate in its development are hyperthermia [6,10] or maternal Insulin Dependent Diabetes Mellitus (IDDM) [6,11], but none of the above have been confirmed to date by epidemiological studies. However, few studies have taken place on the influence of teratogenic substances, such as boric acid, on lab animals [6,12,13] during pregnancy. Hence, their effect on the formation of vertebral elements has not been adequately studied; this would undoubtedly constitute a very important future field of study that could lead to their prevention.

Various genetic factors have also been implicated in the development of congenital scoliosis. Using chromosomal deletion mapping, a large number of chromosomal deletions, relating to regions 2p13 – 15, 6q13 and 15q12 have been identified, leading to the conclusion that said regions play a very important role in its development [6,14].

There are very few reports of congenital scoliosis in monozygotic twins. In specific, only twelve cases have been described in international literature to date and in only four of these did congenital scoliosis appear in both twins [15-23]. In these cases, this genetic origin may not be the only etiologic factor as these may include factors influencing the intrauterine environment, such as hypoxia, the administration of chemical substances or the mother's diet, which lead to disturbances in segmental blood flow to the vertebrae, causing congenital spinal abnormalities. Junghans [24] and Tanaka [25] noted the importance of the distribution of the inter-segmental arteries for accurate vertebral body development. Furthermore Tanaka et al [25] suggests that vascularization process plays an important role in formation of the vertebral body during the stage of resegmentation and early chondrification. Any abnormal distribution of arteries may induce a malformation. This specific hypothesis could explain why, albeit genetically identical, monozygotic twins develop scoliosis at different vertebral levels, as in the currently reported case. This happens because the above factors affect blood flow at different stages of fetal development, thus affecting a different vertebral level.

In addition, theories on disturbances in somite formation or failure in the creation of cartilage in the mesenchymatous tissue try to explain the influence of environmental factors during the intrauterine stage. Somitogenesis describes the process of the formation of the spinal canal's structural elements. In specific, between the 20^th ^and 30^th ^day of embryogenesis, the mesenchymatous cells located paraxially of the position of the future formation of the spinal cord create spherical segmental structures, called somites. Somites are transient embryonic structures that are precursors of the most obvious examples of segmentation in vertebrates: the vertebrae and the intervertebral discs of the backbone, the rib cage and the dermis and striated muscle of the back [26], and they form rhythmically from the prosomitic mesoderm at a time period that is characteristic of the species, ranging from 90 minutes in chicken, 120 minutes in mouse to approximately 4 – 5 hours in humans [27]. The somites' migration and division, which results in the creation of fetal sclerotomes leads to the creation of vertebrae. The overall process appears to be influenced by the cyclic expressed genes participating in the Notch signaling pathway such as Lfng (lunatic fringe), which is a glycosyl-transferase that modifies the Notch receptor, Hes 1, Hes 5, Hes 7 and Hey1 in mice. Various mutations affecting either core Notch members (such as Notch1 gene or Notch coactivator RBKJk) or several oscillator Notch pathway genes, lead to disturbances in the formation of somites and abnormalities in the vertebral elements of lab animals. Wnt/β-catenin pathway via Axin 2 regulates negatively the above process. Moreover, the Wnt 3a gene is necessary for generation of the posterior portion of neuraxis, as knockout mice fail to develop a tailbud and are truncated from a point slightly anterior to the hind limbs. [26-32] Although Wnt3a has been proposed to be a controlling gene in the oscillation of Notch signaling, mutations in the above gene do not appear to be a common reason for the development of congenital vertebral malformations in humans [1].

The assumed influence of environmental factors is reinforced by clinical cases of congenital scoliosis in only one of two monozygotic twins. In particular, in monozygotic twins with identical hair, skin and eye color, ear shape and iridal patterns, and a large numbers of blood group subtypes, only one would manifest congenital scoliosis that would often be accompanied by abnormalities in other systems, such as large ventricular septal defects. The other, on the contrary, would be absolutely healthy [15,18].

The participation of developmental abnormalities from other organs is extremely frequent. We should hence investigate possible anomalies in the cardiovascular, genitourinary and gastrointestinal systems. Cardiovascular system anomalies coexist at a percentage of over 25%; these may be quite mild or even severe, such as the tetralogy of Fallot, Atrial or Ventricular Septal Defects or Transposition of the great vessels. At high percentages of over 20% and up to 43%, congenital scoliosis may be accompanied by genitourinary abnormalities. This could be due to the fact that the genitourinary system and the spinal canal originate in the fetal mesoderm and develop during the fifth week of pregnancy. Ectopic, solitary or lamelliform kidney, ureter duplication and hypospadia cases have been reported. From the gastrointestinal system, the accompanying clinical findings include tracheooesophageal fistulas and esophageal atresia [2,4,8,33].

Nervous system anomalies may also be present at percentages of up to 35%; these include diastematomyelia, Chiari malformation and intradural lipomas. At this point, we should note that the administration of folic acid may be linked to the prevention of the above disorders [2,6,34]. Coexistent musculoskeletal abnormalities, such as Developmental Dysplasia of the Hip, cavus feet and clubfeet or rib cage deformities are not uncommon. Skin participation has also been reported. Pathological pigmentation tests, such as café-au-lait spots, hairy patches and skin tags in the subcutaneous tissue of the scoliotic spinal cord region are necessary. The simultaneous presence of syndromes such as Sprengel Deformity, Klippel-Feil syndrome, Goldenhar's syndrome (craniofacial disorders, microtia, and epibulbar dermoids), Allagile syndrome, Jarcho - Levin syndrome or VACTREL association (Vertebral malformation, Cardiac malformations, TracheoEsophageal fistula, Renal Radial anomalies and Limb defects) require immediate identification and appropriate therapeutic management in these patients.

This is why the diagnostic approach is of such importance. Simple X-rays remain the most reliable indicator not only for the diagnosis but also for the follow-up of these patients [2,3]. At this point, we should note that congenital scoliosis directly influences the respiratory function, as it leads to an increasing asymmetry in lung size. According to a study by Redding, Song et al, there is no correlation between the Cobb angle and pulmonary functionality, so the most reliable method of measuring pulmonary asymmetry is not the measurement of the Cobb angle but a ventilation lung scan [35].

In patients scheduled to undergo corrective operation, the pre-operative use of CT-3D and cardiac U/S is deemed important. In the event of suspected genitourinary abnormalities, the most reliable diagnostic method involves U/S tests. In cases of neurological symptomatology, spinal cords MRIs have widely replaced myelograms in identifying spinal dysraphism [2].

The present paper reports the case of two monozygotic twins, which, albeit genetically identical, present scoliosis at different vertebral levels and with differing deformation grades. This fact, together with the review of the available literature, is added to existing speculation on the extent of influence of genetic and environmental factors during intrauterine life in the development of congenital scoliosis.

## Consent

Written patient consent was obtained for publication of the report.

## Competing interests

The authors declare that they have no competing interests.

## Authors' contributions

TBG built the structure of the paper, performed part of the literature review and revised the manuscript. HRW examined, diagnosed and performed the clinical and X-ray evaluation of the presenting cases as well as part of the literature review and revised the manuscript. AK performed part of the literature review and text editing. All authors have read and approved the final manuscript.
